# Prognosis of Patients with Hypertrophic Obstructive Cardiomyopathy: A Multicenter Cohort Study with Data-Driven Propensity Score Matching Analysis

**DOI:** 10.31083/j.rcm2409267

**Published:** 2023-09-21

**Authors:** Ye He, Huihui Ma, Nian Sun, Shengzhi Zeng, Yanru Zhang, Yan Shu, Wei Hua, Tao Zhou, Ling Zhou, Xiaoping Li

**Affiliations:** ^1^Visual Computing and Virtual Reality Key Laboratory of Sichuan Province, Sichuan Normal University, 610066 Chengdu, Sichuan, China; ^2^Department of Cardiology, Sichuan Provincial People's Hospital, University of Electronic Science and Technology of China, 610072 Chengdu, Sichuan, China; ^3^Department of Cardiology, Chinese Academy of Sciences Sichuan Translational Medicine Research Hospital, 610072 Chengdu, Sichuan, China; ^4^The First Clinical Institute, Zunyi Medical University, 563000 Zunyi, Guizhou, China; ^5^Department of Cardiology, Guanghan People's Hospital, 618300 Guanghan, Sichuan, China; ^6^College of Computer Science and Engineering, University of Electronic Science and Technology of China, 611731 Chengdu, Sichuan, China; ^7^Cardiac Arrhythmia Center, State Key Laboratory of Cardiovascular Disease, Fuwai Hospital, National Center for Cardiovascular Diseases, Chinese Academy of Medical Sciences and Peking Union Medical College, 100037 Beijing, China; ^8^Center of Statistical Research and School of Statistics, Southwestern University of Finance and Economics, 611130 Chengdu, Sichuan, China

**Keywords:** hypertrophic obstruction cardiomyopathy, all-cause mortality, cardiovascular mortality/cardiac transplantation, sudden cardiac death, data-driven propensity score matching

## Abstract

**Background:**

Hypertrophic obstructive cardiomyopathy (HOCM) patients are reported to have a potential risk of sudden cardiac death (SCD); however, HCM with left ventricular outflow tract (LVOT) obstruction, which is regarded as a risk indicator of SCD, is doubtful since the LVOT gradient is dynamic and may be confounded by various environmental factors and routine activities. The purpose of this study was to explore the clinical prognosis of HOCM through a multicenter cohort study with data-driven propensity score matching (PSM) analysis.

**Methods:**

The cohort included 2268 patients with HCM from 1996 to 2021 in 13 tertiary hospitals. In the present study, we excluded 458 patients who underwent alcohol septal ablation (ASA) and septal myectomy (SM) surgery so 1810 HCM patients were eventually included. We developed a data-driven propensity score using 24 demographic and clinical variables to create 1:1 propensity-matched cohorts. A Cox proportional hazard regression model was constructed to assess the effect of HOCM on mortality.

**Results:**

After logit-matching, there were no significant differences in all-cause mortality (log-rank χ^2^ = 1.509, *p* = 0.22), cardiovascular mortality/cardiac transplantation (log-rank χ^2^ = 0.020, *p* = 0.89) or SCD (log-rank χ^2^ = 0.503, *p* = 0.48) between patients with HOCM and hypertrophic nonobstructive cardiomyopathy (HNCM), and according to the Cox proportional hazard regression model, LVOT obstruction was not a risk predictor in patients with HCM.

**Conclusions:**

In both matched and unmatched cohorts, there were no significant differences in clinical prognosis between HOCM and HNCM patients, and LVOT obstruction was not an independent risk predictor of prognosis in patients with HCM.

**Clinical Trial Registration:**

ChiCTR1800017330.

## 1. Introduction

Hypertrophic cardiomyopathy (HCM) is characterized by increased thickness of the left ventricular wall that cannot be explained by abnormal loading conditions (such as hypertension or valvular disease) [1]. Most patients remain asymptomatic or mildly symptomatic throughout their lives, while others have dyspnea, exercise intolerance, chest pain, palpitations, presyncope, and syncope [2, 3]. Clinically, HCM can be classified into 3 types-obstructive, nonobstructive and liable obstructive based on echocardiographic measurement of the difference in peak pressure step between the left ventricular outflow tract (LVOT) gradient [1, 4]. Hypertrophic obstructive cardiomyopathy (HOCM), defined as a maximal LVOT gradient greater than or equal to 30 mmHg at rest or with provocation, is present in approximately two-thirds of patients with HCM [2].

Previous studies have shown that HOCM is an independent predictor of poor prognosis in patients with HCM [5, 6]. However, LVOT obstruction has some unique limitations as a potential risk indicator for sudden cardiac death (SCD), since HCM gradients are dynamic and can be influenced by a variety of environmental factors and routine activities; furthermore, data on the effect of LVOT gradient on the incidence of SCD in HCM patients are rather conflicting [7, 8]. It has also been reported that hypertrophic nonobstructive cardiomyopathy (HNCM) is not always considered to be at low risk [2]. The purpose of this study was to explore the clinical prognosis of HOCM patients through a multicenter cohort study with data-driven propensity score matching (PSM) analysis.

## 2. Methods

### 2.1 Study Population and Diagnostic Criteria

We conducted a multicenter cohort study of 2268 patients with HCM from 13 tertiary hospitals between 1996 and 2021. After excluding 458 patients undergoing alcohol septal ablation (ASA) and septal myectomy (SM) surgery, a total of 1810 patients were fully observed in the study, which included 1263 HNCM patients and 547 HOCM patients.

A data-driven PSM method was used to adjust for potential confounding factors in the comparison of patients with HOCM and HNCM. In particular, our proposed method consisted of two steps. First, instead of using several popular variables from the literature, the propensity score model initially included 24 demographic and clinical variables as much as possible based on the data in a logistic regression model. Second, to avoid overfitting, a data-driven logit-matched method was developed to choose the statistically significant variables in the logistic regression model. Few articles have studied how to choose the variables calculating the propensity score [9, 10], and the most commonly used method was choosing the statistically significant variables in the Cox regression model, namely, the Cox-matched method.

However, those variables are potential risk predictors of mortality based on the Cox regression model. Nevertheless, they may not be important/significant in the PSM model. For fairness of comparison, we added the conventional Cox-matched method in the supplementary materials and demonstrated the applicability of our proposed data-driven logit-matched method.

The patients were diagnosed with HCM by echocardiography or cardiac magnetic resonance (CMR), as a left ventricular (LV) wall thickness ≥15 mm or ≥13 mm in the presence of a first-degree family member affected by HCM. HOCM patients with a maximal LVOT gradient ≥30 mmHg at rest and/or 50 mmHg after provocation and HNCM was opposite to HOCM [11]. Patients with heart or systemic disease capable of developing similar magnitudes of hypertrophy, such as fabry disease, noonan syndrome and amyloidosis cardiomyopathy, were excluded.

### 2.2 Follow-Up and Definitions

The first follow-up began in October 2011, and the last follow-up was completed in May 2022. The first endpoint of the study was all-cause mortality, and the secondary endpoints were cardiovascular mortality/cardiac transplantation and SCD. Cardiovascular mortality was defined as stroke, cerebral infarction, heart failure (HF), and appropriate implantable cardioverter-defibrillator (ICD) discharges. SCD, in which patients who had previously shown a relatively stable or uneventful clinical course died within 1 hour after onset of symptoms or without symptoms. Data on all-cause mortality, cardiovascular mortality/cardiac transplantation and SCD at follow-up were collected by reviewing medical records (outpatient clinic attendance and hospitalization), conducting telephone interviews and reviewing survival status records through the National Police Stations. Patients who lost contact 6 months after discharge were considered lost to follow-up. The hospital’s Institutional Review Board Committee approved the study protocol.

### 2.3 Statistical Analysis

Summary statistics are presented in terms of means ± standard deviations for continuous variables and counts and proportions for categorical variables. Baseline differences between the HOCM and HNCM groups were assessed using the Mann-Whitney tests (Wilcoxon Rank tests) for continuous variables and Pearson chi-square test for categorical variables. The propensity score was calculated to control for variable imbalance between the HOCM and HNCM groups via a logit-matched method.

In the logit-matched method, a logistic regression model was built based on 24 baseline variables. Only those variables with a *p* ≤ 0.1 were then added into the PSM model. Consequently, the propensity score is calculated based on the following 16 variables: sex, New York Heart Association (NYHA) class, atrial fibrillation (AF), non-sustained ventricular tachycardia (NSVT), syncope, log N-terminal fragment pro-brain natriuretic peptide (log NT-pro-BNP), QTc duration, left ventricular (LV) diameter, left atrium (LA) diameter, right ventricular (RV) diameter, left ventricular ejection fraction (LVEF), apical HCM (AHCM), maximal wall thickness, creatinine, beta blockers, and ${\mathrm{Ca}}^{2+}$ antagonists. We matched 1 patient in the HNCM group to each patient in the HOCM group within a small tolerance (0.1 standard deviations of the logit of the propensity score) using the nearest neighbor method, which yielded 484 subjects with HOCM and matched 484 patients with HNCM.

A stepwise variable selection procedure for Cox’s proportional hazard model was applied to find potential risk factors for all-cause mortality, cardiovascular mortality/cardiac transplantation, and SCD in the matched population. Those variables with *p* < 0.05 were considered statistically significant.

Survival curves and their corresponding confidence intervals were estimated by the Kaplan-Meier method, and differences were assessed by the log-rank test. Besides, subgroup analyses were designed based on multiple Cox regression to compare whether HOCM was significant in different indicator subset. Such as sex, age, AF, LV diameter, LVEF, interventricular septum (IVS) thickness, etc. Some indicators of SCD were not analyzed due to the limited mortality. Analyses were performed with R Version 4.1.3 (https://www.r-project.org, the CRAN Mirror: https://mirrors.tuna.tsinghua.edu.cn/CRAN/). Details regarding the Cox-matched method are described in the **Supplementary Material**.

## 3. Results

### 3.1 Baseline Characteristics

There were 1810 patients included in the study, 1263 HNCM patients and 547 HOCM patients. Table 1 summarizes the baseline clinical characteristics of these patients. Compared to HNCM in the unmatched cohort, HOCM had a higher proportion of males and more syncope history, longer QTc and PR duration, smaller LV diameter, larger LA diameter, higher LVEF, maximal wall thickness and IVS thickness, higher level of creatinine and log (NT-pro-BNP), more beta blockers, ${\mathrm{Ca}}^{2+}$ antagonists and less AHCM. The logit-matched cohort analysis showed that 24 baseline variables were not significantly different between HOCM and HNCM (Table 1).

**Table 1.** **Baseline characteristics of HOCM and HNCM groups under the Logit-matched cohort**.

Variables		Unmatched (n = 1810)			% Missing	Matched (n = 968)		
		HNCM	HOCM	*p*-value		HNCM	HOCM	*p*-value
		(n = 1263)	(n = 547)			(n = 484)	(n = 484)	
Female		425 (33.7)	256 (46.8)	<0.001***	0.00	223 (46.1)	219 (45.2)	0.847
Age		57.69 ± 14.47	56.29 ± 15.47	0.096	0.00	57.04 ± 15.31	56.96 ± 15.30	0.847
NYHA classes, I–II, n (%)		891 (70.6)	320 (58.5)	<0.001***	0.05	303 (62.6)	299 (61.8)	0.842
Ventricular arrhythmia, n (%)		230 (18.2)	94 (17.2)	0.648	0.00	72 (14.9)	84 (17.4)	0.336
Atrial fibrillation, n (%)		258 (20.4)	102 (18.6)	0.420	0.00	93 (19.2)	97 (20.0)	0.808
LBBB, n (%)		22 (1.7)	10 (1.8)	1.000	0.00	10 (2.1)	9 (1.9)	1.000
NSVT, n (%)		97 (7.9)	26 (4.9)	0.029*	3.09	20 (4.1)	25 (5.2)	0.541
Syncope, n (%)		122 (9.7)	92 (16.8)	<0.001***	0.00	78 (16.1)	77 (15.9)	1.000
FHCM, n (%)		96 (7.6)	48 (8.8)	0.454	0.05	44 (9.1)	42 (8.7)	0.910
Electrocardiograph								
	QRS, ms	101.46 ± 22.67	105.03 ± 28.69	0.082	15.64	102.88 ± 22.42	104.11 ± 26.22	0.537
	QTc, ms	443.25 ± 44.97	453.67 ± 45.43	<0.001***	17.24	450.44 ± 38.49	450.91 ± 41.39	0.875
	PR, ms	169.14 ± 38.86	175.98 ± 86.77	0.023*	24.25	172.09 ± 38.32	170.94 ± 30.40	0.303
Echocardiography								
	LV diameter, mm	45.59 ± 6.59	42.99 ± 6.70	<0.001***	9.28	43.46 ± 5.51	43.71 ± 6.22	0.485
	LA diameter, mm	39.67 ± 7.16	40.44 ± 7.16	0.012*	8.34	40.08 ± 7.09	40.36 ± 6.97	0.379
	RV diameter, mm	20.00 ± 3.12	20.02 ± 3.10	0.556	13.48	19.84 ± 2.89	19.98 ± 2.88	0.134
	LVEF, %	65.06 ± 10.10	67.14 ± 8.86	<0.001***	10.11	66.55 ± 8.22	66.68 ± 8.40	0.927
	IVS, mm	16.91 ± 4.52	19.26 ± 5.19	<0.001***	7.62	18.56 ± 4.31	18.61 ± 4.61	0.701
	Maximal wall thickness, mm	18.05 ± 4.13	20.37 ± 5.13	<0.001***	6.24	19.57 ± 4.01	19.72 ± 4.54	0.998
	AHCM, n (%)	196 (15.5)	8 (1.5)	<0.001***	0.00	8 (1.7)	8 (1.7)	1.000
Laboratory detection								
	Log (NT-pro-BNP), fmol/L	3.09 ± 0.56	3.23 ± 0.54	<0.001***	27.18	3.20 ± 0.48	3.19 ± 0.48	0.805
	Creatinine, mmol/L	92.91 ± 87.86	81.45 ± 34.50	0.006**	5.80	82.69 ± 36.30	82.72 ± 34.85	0.820
Medicine at baseline								
	Beta blockers, n (%)	901 (71.5)	466 (85.7)	<0.001***	0.28	402 (83.1)	407 (84.1)	0.729
	${\mathrm{Ca}}^{2+}$ antagonists, n (%)	234 (18.6)	158 (29.2)	<0.001***	0.72	124 (25.6)	132 (27.3)	0.610
	ICD, n (%)	34 (2.7)	10 (1.8)	0.349	0.00	16 (3.3)	10 (2.1)	0.321

Abbreviations: NYHA, New York Heart Association; LBBB, left bundle branch block; NSVT, non-sustained ventricular tachycardia; LV, left ventricular; LVEF, left ventricular ejection fraction; LA, left atrium; RV, right ventricular; IVS, interventricular septum; AHCM, apical HCM; FHCM, familial HCM; NT-pro-BNP, N-terminal fragment pro-brain natriuretic peptide; ICD, implantable cardioverter defibrillator; HNCM, hypertrophic nonob-structive cardiomyopathy; HOCM, hypertrophic obstructive cardiomyopathy. Note: “***” represent the significant level *p* ≤ 0.001, “**” represent the significant level *p* ≤ 0.01, “*” represent the significant level *p* < 0.05.

**Supplementary Table 1** in the supplementary material shows the matching results of the Cox-matched cohort analysis; however, the data of history of syncope, IVS thickness and maximal wall thickness were significantly different between HOCM and HNCM after matching regardless of the primary or the secondary endpoint. The matching results showed that our proposed data-driven logit-matched method outperformed the conventional Cox-matched method in terms of successfully matching proportions.

### 3.2 Follow-up Results of the Unmatched Cohort

**Fig. 1.**
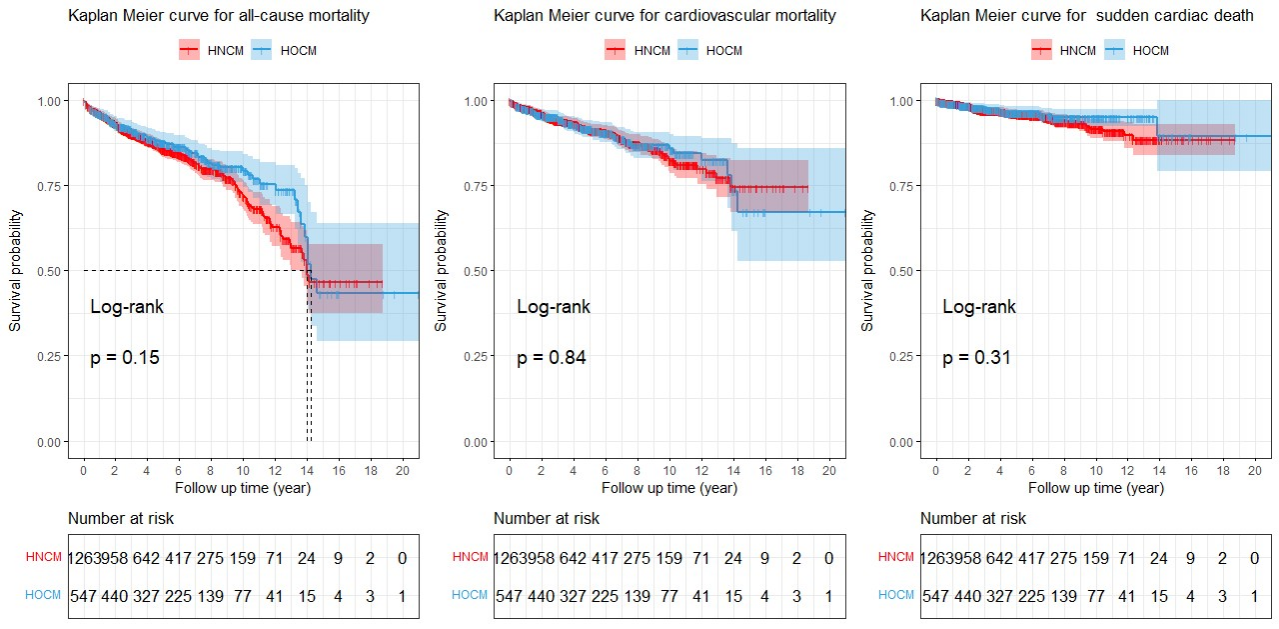
**Kaplan-Meier curves for the unmatched cohort**. HNCM, hypertrophic nonobstructive cardiomyopathy; HOCM, hypertrophic obstructive cardiomyopathy.

The Kaplan-Meier curves for the unmatched cohort are presented in Fig. 1. In the unmatched cohort during a mean follow-up time of 5.2 ± 3.8 years, 303 all-cause mortalities occurred in the current analysis (87 deaths among HOCM patients and 216 deaths among HNCM patients). A total of 118 were cardiovascular mortalities in HNCM and 55 in HOCM (9.3% versus 10.1%), while there were 54 SCD in HNCM and 20 SCD in HOCM (4.3% versus 3.7%). The Kaplan-Meier curves analysis showed that there were no significant differences between HOCM and HNCM in all-cause mortality (log-rank χ^2^ = 2.034, *p* = 0.15), cardiovascular mortality/cardiac transplantation (log-rank χ^2^ = 0.041, *p* = 0.84) or SCD (log-rank χ^2^ = 1.012, *p* = 0.31) before match.

### 3.3 Follow-up Results of the Matched Cohort

#### 3.3.1 Primary Outcome: All-Cause Mortality

**Fig. 2.**
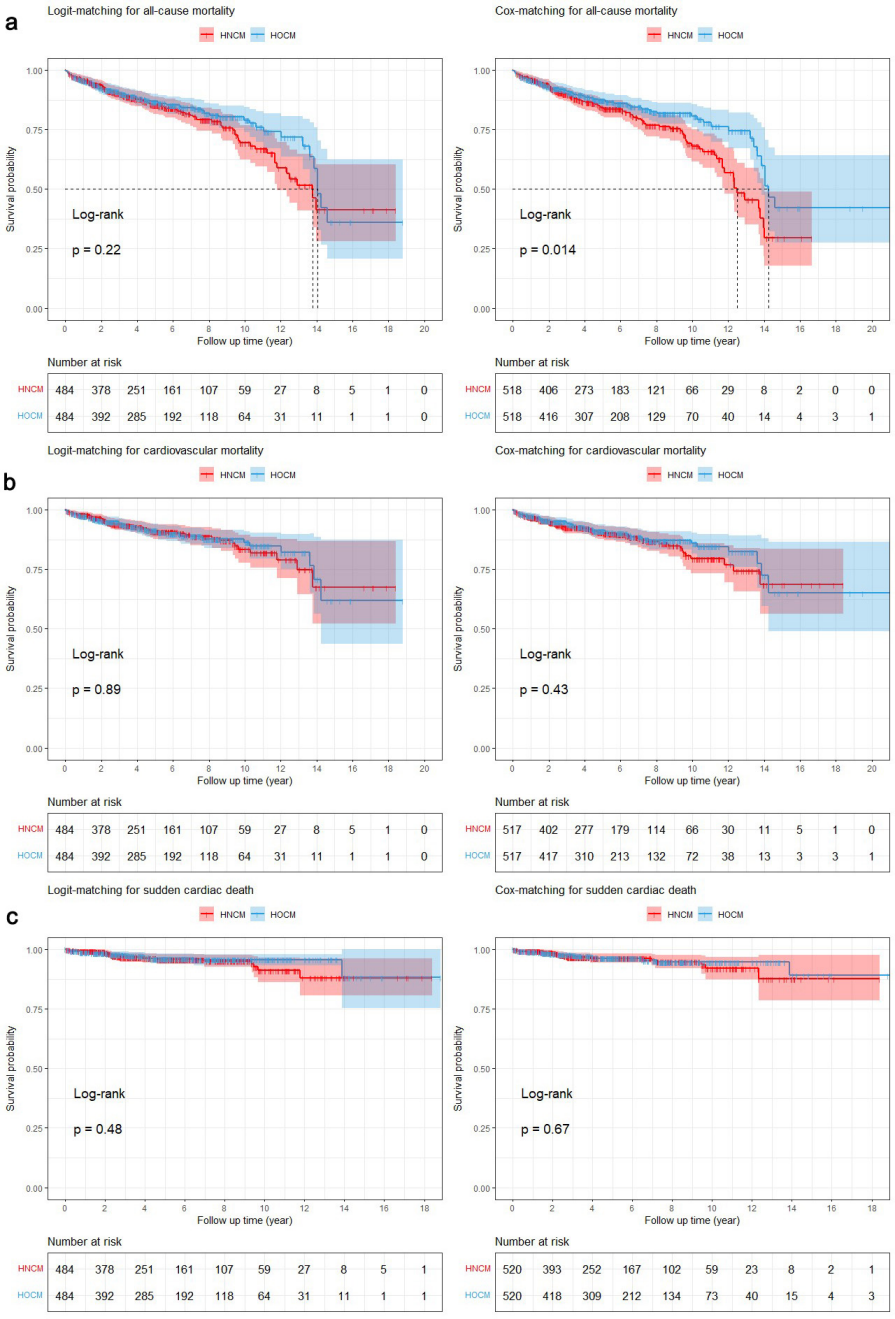
**Kaplan-Meier curves for Logit-matching cohort and Cox-matching cohort in all-cause mortality, cardiovascular mortality/cardiac transplantation and SCD**. (a) All-cause mortality. (b) Cardiovascular mortality/cardiac transplantation. (c) SCD. HNCM, hypertrophic nonobstructive cardiomyopathy; HOCM, hypertrophic obstructive cardiomyopathy; SCD, sudden cardiac death.

After logit matching, there was no significant difference between the Kaplan-Meier curves of HOCM and HNCM in all-cause mortality, but the inverse was true in the Cox-matched cohort (logit-matched: log-rank χ^2^ = 1.509, *p* = 0.22; Cox-matched: log-rank χ^2^ = 6.018, *p* = 0.014) (Fig. 2a).

According to the Cox proportional hazard regression model, LVOT gradient was not a predictor of all-cause mortality (Table 2a). In the logit-matched cohort, age [hazard ratio (HR): 1.023; 95% CI: 1.012–1.035; *p* < 0.001], NYHA I-II class [HR: 0.640; 95% CI: 0.468–0.877; *p* = 0.006], LV diameter [HR: 0.696; 95% CI: 0.952–0.987; *p* = 0.023], LVEF [HR: 0.696; 95% CI: 0.952–0.987; *p* < 0.001] and log (NT-pro-BNP) [HR: 4.776; 95% CI: 3.492–6.532; *p* < 0.001] were risk factors for all-cause mortality.

**Table 2a.** **Multivariate Cox regression for all-cause mortality**.

Variables	Logit-matched cohort			Cox-matched cohort		
	Hazard ratio	95% CI	*p*-value	Hazard ratio	95% CI	*p*-value
Obstruction	—	—	—	0.778	(0.580–1.044)	0.094
Age	1.023	(1.012–1.035)	<0.001***	1.026	(1.015–1.038)	<0.001***
NYHA I-II class	0.640	(0.468–0.877)	0.006**	0.776	(0.578–1.042)	0.091
AF	0.688	(0.473–1.002)	0.051	0.742	(0.522–1.055)	0.096
LVEF	0.969	(0.952–0.987)	<0.001***	0.968	(0.953–0.983)	<0.001***
Log (NT-pro-BNP)	4.776	(3.492–6.532)	<0.001***	4.319	(3.159–5.905)	<0.001***
LV diameter	0.969	(0.942–0.996)	0.023*	—	—	—
Concordance	0.755			0.747		

Note: NYHA, New York Heart Association; NT-pro-BNP, N-terminal fragment pro-brain natriuretic peptide; CI, confidence interval; AF, atrial fibrillation; LVEF, left ventricular ejection fraction; LV, left ventricular. “***” represent the significant level *p* ≤ 0.001; “**” represent the significant level *p* ≤ 0.01; “*” represent the significant level *p* < 0.05; “—” indicates that there is no value.

#### 3.3.2 Secondary Outcomes: Cardiovascular Mortality/Cardiac Transplantation and SCD

There was no significant difference between the Kaplan‒Meier curves of the two matched groups in cardiovascular mortality/cardiac transplantation (logit-matched, log-rank χ^2^ = 0.020, *p* = 0.89; Cox-matched, log-rank χ^2^ = 0.615, *p* = 0.43) (Fig. 2b). The Cox regression model is presented in Table 2b. The LVOT gradient did not predict cardiovascular mortality/cardiac transplantation in the logit-matched cohort. Specifically, in the logit-matched cohort, age [HR: 1.016; 95% CI: 1.001–1.031; *p* = 0.034], NYHA I-II class [HR: 0.565; 95% CI: 0.373–0.857; *p* = 0.007], LVEF [HR: 0.971; 95% CI: 0.949–0.993; *p* = 0.011] and log (NT-pro-BNP) [HR: 3.546; 95% CI: 2.308–5.450; *p* < 0.001] were risk factors for cardiovascular mortality/cardiac transplantation.

**Table 2b.** **Multivariate Cox regression for cardiovascular mortality/cardiac transplantation**.

Variables	Logit-matching			Cox-matching		
	Hazard ratio	95% CI	*p*–value	Hazard ratio	95% CI	*p*-value
NYHA I-II class	0.565	(0.373–0.857)	0.007**	0.654	(0.446–0.960)	0.030*
LVEF	0.971	(0.949–0.993)	0.011*	0.968	(0.949–0.988)	0.002**
Log (NT-pro-BNP)	3.546	(2.308–5.450)	<0.001***	4.180	(2.775–6.297)	<0.001***
RV diameter	0.947	(0.882–1.016)	0.131	0.949	(0.892–1.010)	0.098
Age	1.016	(1.001–1.031)	0.034*	—	—	—
Concordance	0.733			0.744		

Note: NYHA, New York Heart Association; NT-pro-BNP, N-terminal fragment pro-brain natriuretic peptide; CI, confidence interval; LVEF, left ventricular ejection fraction; RV, right ventricular. “***” represent the significant level *p* ≤ 0.001; “**” represent the significant level *p ≤* 0.01; “*” represent the significant level *p* < 0.05; “—” indicates that there is no value.

The Kaplan-Meier curve for SCD is presented in Fig. 2c, and the results from the Cox model are presented in Table 2c. After matching, there was no significant difference between the Kaplan-Meier curves of the two groups (logit-matched, log-rank χ^2^ = 0.503, *p* = 0.48; Cox-matched, log-rank χ^2^ = 0.178, *p* = 0.67), and the LVOT gradient did not predict mortality. In particular, in the logit-matched cohort, age [HR: 0.976; 95% CI: 0.956–0.997; *p* = 0.022], LA diameter [HR: 1.050; 95% CI: 1.004–1.097; *p* = 0.032] and log (NT-pro-BNP) [HR: 4.338; 95% CI: 2.137–8.804; *p* < 0.001] were risk factors for SCD.

**Table 2c.** **Multivariate Cox regression for sudden cardiac death**.

Variables	Logit-matching			Cox-matching		
	Hazard ratio	95% CI	*p*-value	Hazard ratio	95% CI	*p*-value
Age	0.976	(0.956–0.997)	0.022*	0.972	(0.954–0.992)	0.005**
QRS	1.010	(1.000–1.021)	0.061	1.010	(1.000–1.020)	0.058
Log (NT-pro-BNP)	4.338	(2.137–8.804)	<0.001***	4.949	(2.418–10.131)	<0.001***
RV diameter	0.912	(0.815–1.020)	0.108	0.902	(0.808–1.007)	0.067
LA diameter	1.050	(1.004–1.097)	0.032*	1.065	(1.019–1.112)	0.005**
Female	0.505	(0.232–1.097)	0.084	—	—	—
Syncope	—	—	—	0.346	(0.083–1.438)	0.144
Concordance	0.785			0.798		

Note: NT-pro-BNP, N-terminal fragment pro-brain natriuretic peptide; CI, confidence interval; RV, right ventricular; LA, left atrium. “***” represent the significant level *p* ≤ 0.001; “**” represent the significant level *p* ≤ 0.01; “*” represent the significant level *p* < 0.05; “—” indicates that there is no value.

### 3.4 Subgroup Analysis

**Fig. 3.**
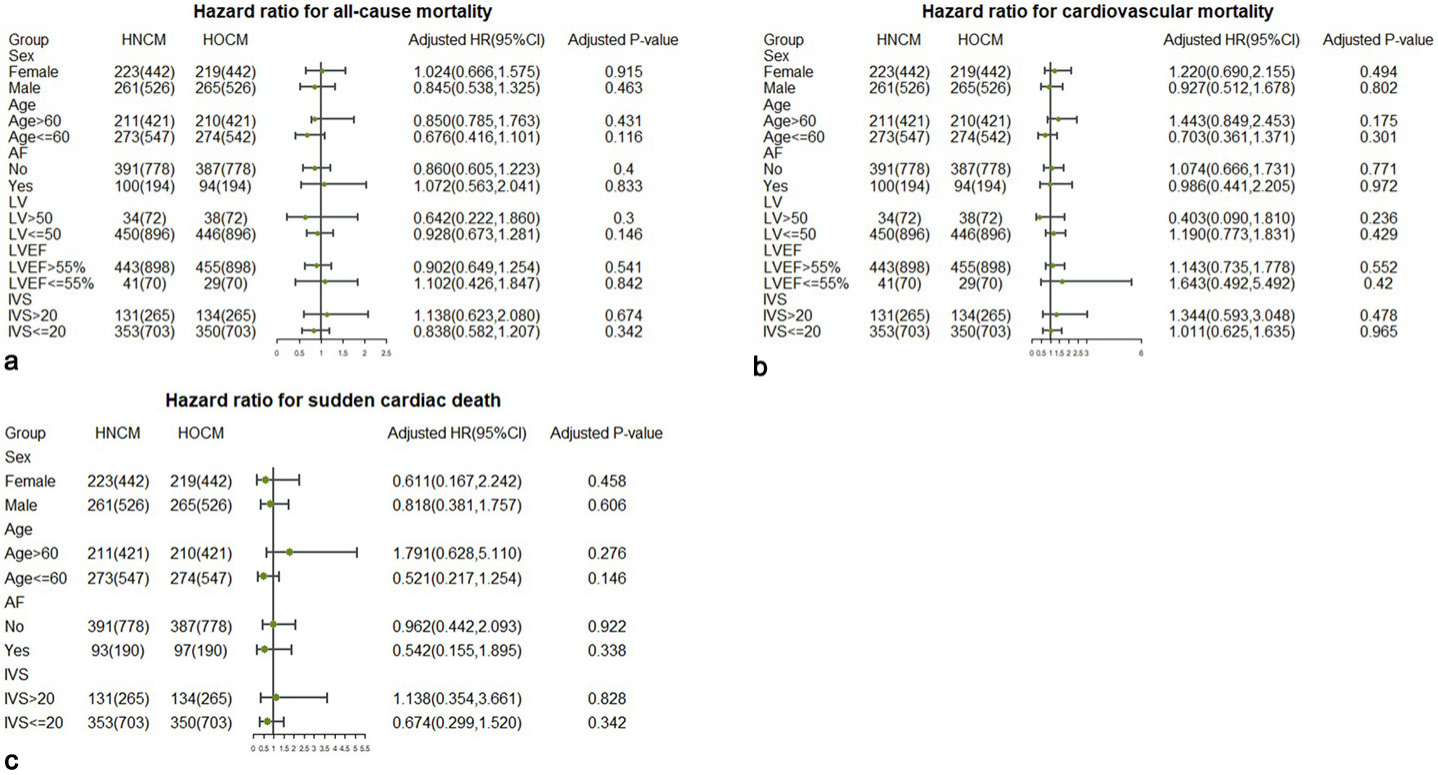
**Forest plots of all-cause mortality, cardiovascular mortality/cardiac transplantation and SCD in subgroup analyses**. (a) All-cause mortality. (b) Cardiovascular mortality/cardiac transplantation. (c) SCD. SCD, sudden cardiac death; AF, atrial fibrillation; LV, left ventricular; LVEF, left ventricular ejection fraction; IVS,interventricular septum; HR, hazard ratio; HNCM, hypertrophic nonob-structive cardiomyopathy; HOCM, hypertrophic obstructive cardiomyopathy.

Subgroup analysis was designed to better compare whether HOCM was significant in different subsets. Fig. 3a–c forest plots show the results of subgroup analyses in all-cause mortality, cardiovascular mortality/cardiac transplantation and SCD, respectively. The results indicated that HOCM was not significant among all subgroups of all-cause mortality, cardiovascular mortality/cardiac transplantation and SCD.

## 4. Discussion

In the present study, we found that the LVOT gradient had no effect on HCM prognosis either before or after matching by data-driven PSM analysis in a multicenter cohort study. Additionally, subgroup analyses showed that HOCM was not significant in all subgroups of all-cause mortality, cardiovascular mortality/cardiac transplantation and SCD.

HOCM occurs in up to 70% of HCM patients and is associated with pernicious events and has been considered a marker of poor prognosis [6, 12]. LVOT obstruction is reported to be associated with symptom progression and increased mortality in HCM patients and is an independent predictor of adverse outcomes, arrhythmias, and SCD [1, 5, 13]. However, the incidence of cardiovascular mortality/cardiac transplantation and SCD in patients with HOCM varies between studies [2, 14]. The long-term prognosis of HOCM has been shown to be similar to that of the general population [4]. Furthermore, Pozios *et al*. [2] found that HNCM patients had 4 times more ventricular tachycardia/ventricular fibrillation episodes than labile-obstructive patients and 3 times more ventricular tachycardia/ventricular fibrillation episodes than HOCM patients. Moreover, ICD discharges were also more frequent in the HNCM subgroups [2]. Similarly, in the present study, ICD implantation was more common in HNCM patients. In addition, another study revealed that only 30% of HCM-related deaths were associated with LVOT obstruction [15]. In patients with HCM with a benign presentation and without risk factors, only 29% with SCD had LVOT obstruction [16]. Therefore, LVOT obstruction alone may not always confer high risk and thus is not considered to be one of the traditional risk factors for SCD [3].

In the present study, to better study the effect of LVOT gradient on the prognosis of HCM, we excluded those patients who were undergoing ASA and SM surgery. Furthermore, a data-driven PSM analysis was performed to adjust for potential confounders from other baseline variables between HOCM and HNCM. And subgroup analysis was performed to study whether HOCM was significant in different clinical factors subsets. The result showed that there were no significant differences in the prognosis of HOCM and HNCM in the logit-matched cohorts, and LVOT obstruction had no impact on the prognosis of HCM. Also, HOCM was not significant in different subsets for all-cause mortality, cardiovascular mortality/cardiac transplantation and SCD. In the Cox-matched cohorts, there was a significant difference in all-cause mortality between HOCM and HNCM, which was in contrast to the pre-matched and logit-matched results. We consider it possible that Cox matching did not match all indicators, so the results may not be reliable. We recommend using the logit-matched method, for which the logit-matched method outperforms the conventional Cox-matched method in terms of successfully matching proportions.

Moreover, LVOT obstruction had no effect on HCM prognosis in the present study. Considering that this study is a retrospective, another reason may be that people with HOCM are more cautious and keep in mind the advice of their physicians to avoid sudden heavy work, which is often a precursor to SCD [17, 18]. On the other hand, HCM gradients are dynamic, spontaneous changes that can be influenced by a variety of environmental factors and daily activities [18]. Additionally, the contribution of LVOT obstruction to risk stratification is considered to be limited due to the low annual rate of SCD and the particularly low positive predictive value of obstruction [19]. Finally, studies have shown that the prognosis of patients with HOCM after clinical treatment was not different from that of age- and sex-matched populations [4, 20].

Historically, cardiomyopathy was the main cause of SCD in young people under 35 years of age [21]. And HCM has been reported to be one of the most common causes of SCD in young children and adults, the annual incidence of SCD in children, adolescents or young adults was 2% and in adults it was 0.5–1.5% [1, 22]. And HOCM was associated with an increased risk of SCD and heart failure [1]. However, most studies have failed to show an association between LVOT gradient and poor prognosis, and only two large studies have shown a slightly increased risk of SCD in patients with a resting gradient ≥30 mmHg [23]. Therefore, the effect of LVOT obstruction on the risk of SCD has been debatable [24, 25, 26], as some HNCM patients produce significant changes in the LVOT gradient even when regular daily activity is performed [7, 27]. The unique ability of patients with HCM to transition briefly from an obstructive to a nonobstructive state alters the full significance of this risk factor in some patients [25]. While the prognostic role of exercise-induced LVOT obstruction is uncertain, it is currently not included in the calculator of SCD risk scores [24]. Therefore, the 2011 American College of Cardiology Foundation/American Heart Association (ACCF/AHA) HCM guidelines only identify LVOT obstruction as a potential moderator of SCD risk and include it in borderline cases [28]. It was not until the 2014 European Society of Cardiology (ESC) HCM guidelines identified LVOT obstruction as the most important clinical feature for increased risk of SCD [11].

## 5. Limitations

There are some limitations in the present study. First, this is a multicenter retrospective study, and the patients included in the study were from 13 tertiary centers, so there may be some heterogeneity among the different hospitals. Second, in the present study we were focusing on the impact of LVOT on HCM mortality, and considering that surgery would change the primary LVOT obstruction of patients, so we excluded patients who underwent ASA and SM. Third, not all patients had a Valsalva maneuver, so there may be liable-obstruction diagnosed as non-obstruction. Fourth, as shown in the ESC HCM risk-SCD calculator, not only the presence or absence of LVOT gradient but also its degree is associated with prognosis. Unfortunately, there were too many missing data of LVOT gradient in the present study, so we did not make further analysis. Finally, the medications were only recorded during the in-hospital treatment of the patients, and no follow-up data were recorded, which we did not further analyzed in the present study.

## 6. Conclusions

In the present multicenter cohort study, there were no significant differences in all-cause mortality, cardiovascular mortality/cardiac transplantation or SCD between HOCM and HNCM before and after matching analysis, and according to the Cox proportional hazard regression model, LVOT obstruction was not an independent risk predictor of HCM.

## Data Availability

All data generated or analyzed during this study are included in this published article.

## Supplementary Material

Supplementary material associated with this article can be found, in the online version, at https://doi.org/10.31083/j.rcm2409267.
