# Supplementary material for: Prognosis of Patients with Hypertrophic Obstructive Cardiomyopathy: A Multicenter Cohort Study with Data-Driven Propensity Score Matching Analysis
Source: Rev Cardiovasc Med. 2023 Sep 21;24(9):267. doi: 10.31083/j.rcm2409267 (PMC11270055; doi:10.31083/j.rcm2409267)
Supplement: Supplementary file 1 [file 2153-8174-24-9-267-s1.zip › 2153-8174-24-9-267-s1.docx]

**Supplementary material**

In the Cox-matched method, to find potential confounding in the comparison between HOCM and HNCM groups under the primary and the secondary endpoints, 23 variables were evaluated in a univariate Cox proportional model, and variables with a P value of 0.1 were then entered into a propensity score matching.

For the primary all-cause mortality endpoint, the underlying variables: male, sex, NYHA classes, age, AF, NSVT, syncope, QRS during, LV diameter, RV diameter, LA diameter, LVEF, log (NT-pro-BNP), creatinine, ventricular arrhythmias were chosen to estimate the propensity score. For the secondary cardiovascular mortality/cardiac transplantation endpoint, the underlying variables: male, AF, NSVT, familial HCM (FHCM), LV diameter, LA diameter, LVEF, log (NT-pro-BNP), ventricular arrhythmias were chosen to estimate the propensity score. Male, NSVT, LV diameter, LVEF were chosen to estimate the propensity score for the secondary SCD endpoint.

We then attempted to caliper match 1 patient in the HNCM group to each patient in the HOCM group within a small tolerance (0.1 standard deviations of the Logit of the propensity score) using the nearest neighbor method, which yielded n=518 for the all-cause mortality, n=517 patients for the cardiovascular mortality/cardiac transplantation, and n=520 patients for the SCD endpoint.

Table-E1. Baseline characteristics of HOCM and HNCM groups under the cox-matched cohort.

| Variables | All-cause mortality  (n=1036) | | |  | Cardiovascular mortality/ cardiac transplantation (n=1034) | | |  | Sudden cardiac death  (n=1040) | | |
| --- | --- | --- | --- | --- | --- | --- | --- | --- | --- | --- | --- |
|  | **HNCM**  **(n=518)** | **HOCM**  **(n=518)** | **P-value** |  | **HNCM**  **(n=517)** | **HOCM**  **(n=517)** | **P-value** |  | **HNCM**  **(n=520)** | **HOCM**  **(n=520)** | **P-value** |
| Male | 250(48.3) | 235(45.4) | 0.383 |  | 237(45.8) | 229(44.3) | 0.662 |  | 205(39.4) | 238(45.8) | 0.045* |
| Age | 57.10±14.76 | 56.69±15.28 | 0.584 |  | 56.93±15.65 | 56.57±15.26 | 0.575 |  | 57.19±14.89 | 56.70±15.13 | 0.635 |
| NYHA classes, I-II, n (%) | 312(60.2) | 310(59.8) | 0.949 |  | 319(61.7) | 310(60.0) | 0.610 |  | 373(71.7) | 304(58.5) | <0.001*** |
| Ventricular arrhythmias, n (%) | 81(15.6) | 92(17.8) | 0.405 |  | 81(15.7) | 88(17.0) | 0.614 |  | 85(16.3) | 88(16.9) | 0.868 |
| Atrial fibrillation, n (%) | 116(22.4) | 102(19.7) | 0.322 |  | 110(21.3) | 98(19.0) | 0.393 |  | 110(21.2) | 100(19.2) | 0.487 |
| LBBB, n (%) | 12(2.3) | 10(1.9) | 0.829 |  | 10(1.9) | 10(1.9) | 1.000 |  | 10(1.9) | 9(1.7) | 1.000 |
| NSVT, n (%) | 29(5.6) | 26(5.0) | 0.782 |  | 37(7.2) | 25(4.8) | 0.150 |  | 37(7.1) | 25(4.8) | 0.150 |
| Syncope, n (%) | 62(12.0) | 89(17.2) | 0.022* |  | 59(11.4) | 90(17.4) | 0.008** |  | 57(11.0) | 86(16.5) | 0.012* |
| FHCM, n (%) | 58(11.2) | 45(8.7) | 0.213 |  | 49(9.5) | 43(8.3) | 0.585 |  | 45(8.7) | 43(8.3) | 0.911 |
| *Electrocardiograph* |  |  |  |  |  |  |  |  |  |  |  |
| QRS, ms | 103.40±22.32 | 104.52±27.16 | 0.886 |  | 103.64±23.01 | 103.85±25.17 | 0.979 |  | 103.82±22.55 | 104.29±26.55 | 0.683 |
| QTc, ms | 449.56±42.65 | 452.07±41.81 | 0.761 |  | 451.28±37.53 | 451.50±41.32 | 0.975 |  | 445.49±43.63 | 452.43±42.12 | 0.065 |
| PR, ms | 170.44±35.62 | 171.79±33.12 | 0.161 |  | 172.06±34.62 | 171.82±32.95 | 0.584 |  | 169.80±34.51 | 171.75±32.27 | 0.165 |
| *Echocardiography* |  |  |  |  |  |  |  |  |  |  |  |
| LV diameter, mm | 44.82±6.32 | 43.31±6.34 | <0.001*** |  | 43.52±5.74 | 43.40±6.23 | 0.714 |  | 43.78±5.65 | 43.47±6.18 | 0.549 |
| LA diameter, mm | 40.56±7.17 | 40.37±6.86 | 0.982 |  | 40.54±7.38 | 40.21±6.66 | 0.675 |  | 40.61±7.12 | 40.29±6.72 | 0.594 |
| RV diameter, mm | 19.95±2.99 | 20.02±2.88 | 0.369 |  | 19.71±3.10 | 20.02±2.83 | 0.025* |  | 19.91±3.06 | 20.01±2.83 | 0.300 |
| LVEF, % | 65.94±9.09 | 66.74±8.42 | 0.212 |  | 66.76±8.39 | 66.88±8.44 | 0.819 |  | 66.52±8.37 | 66.78±8.42 | 0.783 |
| IVS, mm | 17.91±4.33 | 19.08±5.03 | 0.001** |  | 18.19±4.16 | 19.04±4.93 | 0.026* |  | 18.08±4.22 | 19.04±4.99 | 0.008** |
| Maximal wall thickness, mm | 18.84±4.08 | 20.20±4.96 | <0.001*** |  | 18.99±4.01 | 20.18±4.92 | <0.001*** |  | 18.86±4.04 | 20.22±4.99 | <0.001*** |
| AHCM, n (%) | 4(0.8) | 8(1.5) | 0.384 |  | 6(1.2) | 8(1.6) | 0.788 |  | 4(0.8) | 8(1.5) | 0.384 |
| *Laboratory detection* |  |  |  |  |  |  |  |  |  |  |  |
| Log (NT-pro-BNP), fmol/L | 3.21±0.47 | 3.20±0.47 | 0.302 |  | 3.20±0.49 | 3.20±0.48 | 0.454 |  | 3.19±0.46 | 3.19±0.47 | 0.662 |
| Creatinine, mmol/L | 81.82±34.07 | 82.85±33.95 | 0.421 |  | 81.77±30.72 | 82.80±33.94 | 0.561 |  | 82.76±30.44 | 82.71±33.90 | 0.784 |
| *Medicine at baseline* |  |  |  |  |  |  |  |  |  |  |  |
| Beta blockers, n (%) | 444(85.7) | 440(84.9) | 0.792 |  | 370(71.6) | 445(86.1) | <0.001*** |  | 380(73.1) | 449(86.3) | <0.001*** |
| Ca^2+^antagonists, n (%) | 131(25.3) | 138(26.6) | 0.671 |  | 129(25.0) | 138(26.7) | 0.570 |  | 141(27.1) | 144(27.7) | 0.889 |

*Abbreviations: NYHA=New York Heart Association, LBBB=* *left bundle branch block, NSVT=non-sustained ventricular tachycardia, LV=left ventricular, LA=left atrial, RV=right ventricular, IVS= interventricular septum, LVEF=left ventricular ejection fraction, LVOT=left ventricular outflow tract, AHCM=apical HCM, FHCM=familial HCM, NT-pro-BNP=N-terminal fragment pro-brain natriuretic peptide*

*Note: “***” represent the significant level p≤0.001, “**” represent the significant level p≤0.01, “*” represent the significant level p<0.05.*
